# Bird Fancier’s lung: a rare clinical image

**DOI:** 10.11604/pamj.2024.47.85.42406

**Published:** 2024-02-26

**Authors:** Anjana Ledwani, Ashwin Karnan

**Affiliations:** 1 Department of Respiratory Medicine, Jawaharlal Nehru Medical College, Datta Meghe Institute of Higher Education and Research, Sawangi (Meghe), Wardha, Maharashtra, India

**Keywords:** Pigeon breeder’s lung, hypersensitivity pneumonitis, lung opacity, steroid

## Image in medicine

A 19-year-old male college student presented to the Outpatient Department with complaints of dyspnea, dry cough, and cold for the past 1 month not responding to any treatment with no significant past and personal history. On a detailed history, the patient revealed passive smoking and breeding pigeons at his home for the past 3 years. On examination, he was conscious, and oriented, pulse rate of 104 beats/minute, Respiratory rate-20 breaths/minute, oxygen saturation of 96% on room air, blood pressure of 130/80mm Hg, and bilateral fine crepitations on the respiratory system examination. All routine investigations were done. Chest X-ray showed bilateral nonhomogeneous opacities spread in the batwing pattern. High-resolution computed tomography (HRCT) of the thorax was done which was suggestive of bilateral ground glass opacification with centrilobular nodules. Induced sputum was sent for ZN staining, which was negative. Pigeon serum proteins, feathers, and droppings-specific degree of total immunoglobulin G (IgG) testing was done, which was positive. The patient was advised to avoid contact with birds and started on oral glucocorticoids. At 22-weeks follow-up, the Patient showed drastic improvement clinically and radiologically. Bird fancier's lung is a type of hypersensitivity pneumonitis caused by recurrent exposure to avian antigens. Pathophysiology is exposure to antigen causing immune complex formation with the influx of neutrophils leading to inflammation and ultimately fibrosis. Patients usually present with progressing dyspnoea, cough, and chest discomfort. The mainstay of treatment is corticosteroids along with cessation of exposure to the antigens. However, if left untreated, it can lead to fibrosis and irreversible damage.

**Figure 1:**
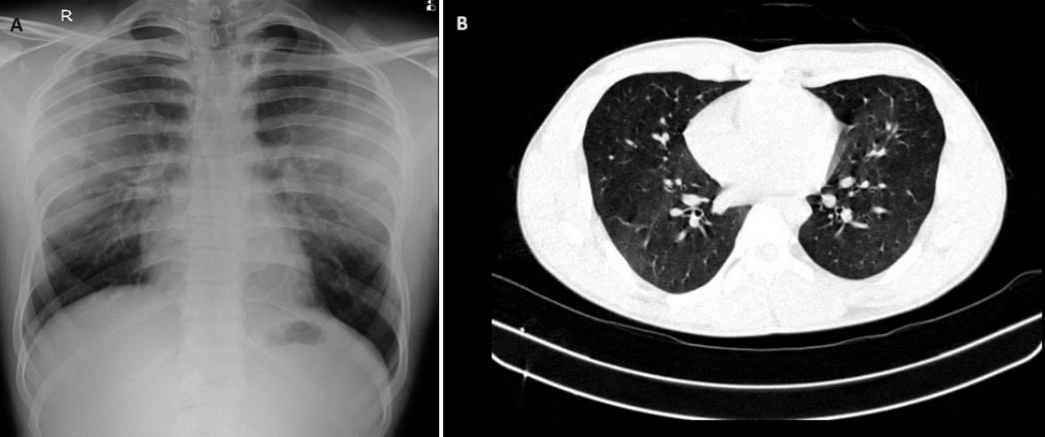
A) chest Xray showing bilateral non homogenous opacity; B) high resolution CT thorax showing bilateral ground glass opacification with centrilobular nodules

